# Impact of Low-Burden *TP53* Mutations in the Management of CLL

**DOI:** 10.3389/fonc.2022.841630

**Published:** 2022-02-08

**Authors:** Gregory Lazarian, Florence Cymbalista, Fanny Baran-Marszak

**Affiliations:** Service d’Hématologie Biologique, Hôpital Avicenne, Assistance Publique des Hôpitaux de Paris, Paris, France

**Keywords:** CLL (Chronic Lymphocytic Leukemia), *TP53*, NGS (next-generation sequencing), clinical impact, minor clone

## Abstract

In chronic lymphocytic leukemia (CLL), *TP53* abnormalities are associated with reduced survival and resistance to chemoimmunotherapy (CIT). The recommended threshold to clinically report *TP53* mutations is a matter of debate given that next-generation sequencing technologies can detect mutations with a limit of detection of approximately 1% with high confidence. However, the clinical impact of low-burden *TP53* mutations with a variant allele frequency (VAF) of less than 10% remains unclear. Longitudinal analysis before and after fludarabine based on NGS sequencing demonstrated that low-burden *TP53* mutations were present before the onset of treatment and expanded at relapse to become the predominant clone. Most studies evaluating the prognostic or predictive impact of low-burden *TP53* mutations in untreated patients show that low-burden *TP53* mutations have the same unfavorable prognostic impact as clonal defects. Moreover, studies designed to assess the predictive impact of low-burden *TP53* mutations showed that *TP53* mutations, irrespective of mutation burden, have an inferior impact on overall survival for CIT-treated patients. As low-burden and high-burden *TP53* mutations have comparable clinical impacts, redefining the VAF threshold may have important implications for the clinical management of CLL.

## Introduction

The heterogeneous clinical course of chronic lymphocytic leukemia has highlighted the need to define prognostic and predictive markers to improve the management of patients (1). On one hand, prognostic markers reflect the underlying biology and natural history of CLL and are informative for untreated patients or those requiring treatment (2, 3). On the other hand, predictive markers provide information on the likely benefits or contraindications of a given treatment. *TP53* abnormalities, namely, both deletion of the 17p chromosome and mutations at *TP53* loci, are one of the gold standards of high risk in CLL because these abnormalities indicate both an adverse prognosis and predict chemoresistance (4, 5). In the past decade, the therapeutic landscape of CLL has considerably improved, offering the possibility for patients with *TP53* defects to benefit from targeted therapy with BcR pathway or bcl2 inhibitors (6–8). Although the first-line treatment strategy may differ among countries, assessment of *TP53* status has become essential, as it serves as a contraindication for the use of chemoimmunotherapy (CIT) (9). Hence, in daily clinical practice, the use of *TP53* status as a predictive marker is mandatory for treatment decisions before the addition of each new line of treatment (10, 11).

The implementation of NGS sequencing technologies with high sensitivity has facilitated the detection of *TP53* mutations with the possibility of detecting variants with allelic fractions (VAFs) below the current conventional threshold of 10% published by the European Research Initiative on Chronic Lymphocytic Leukemia (ERIC) in 2018 (12), above which *TP53* mutations should be clinically reported. Nevertheless, the clinical and biological relevance of these minor clones is debated.

The definition of minor clones and their biological and clinical significance have been discussed in numerous studies. However, contradictory results are often reported that might be in part attributed to different cohort compositions and variable low-burden threshold definitions. To clarify the clinical role of low-burden *TP53* mutations in CLL, the prognostic and predictive impact of *TP53* mutations were analyzed in different cohorts. The results and conclusions are discussed in this review.

## What is a Low-Burden *TP53* Mutation or minor Clone?

Del(17p) associated with *TP53* mutations is the most common abnormality affecting the *TP53* gene in CLL, accounting for approximately two-thirds of cases. The remaining cases either exclusively harbor *TP53* gene mutation(s) or rarely a 17p deletion. Moreover, *TP53* mutation can be accompanied by the mutation of the second allele or a copy number neutral loss of heterozygosity (13).

Historically, *TP53* abnormalities were first analyzed by conventional karyotyping combined with Fluorescence *In Situ* Hybridization (FISH), which allowed the detection of cells carrying a deletion of chromosome 17p13.1 (TP53) with a sensitivity of >5% positive cells (14). Despite a relatively good sensitivity of detection, cytogenetic techniques failed to detect approximately 30–40% of patients carrying only mutations in the gene. Later, *TP53* mutation screening relied on Sanger sequencing covering exons 4 to 9 of the gene with a sensitivity of approximately 10–20%. Hence, combining FISH analysis and sequencing substantially improved the detection of *TP53* aberrations. The advent of NGS technologies next provided the opportunity to reduce the threshold of detection of *TP53* mutations and to deeply examine the clonal heterogeneity of CLL. In a retrospective analysis of newly diagnosed patient samples, NGS sequencing could detect low-burden *TP53* mutations previously identified as unmutated by Sanger sequencing due to their low abundance in the tumor cell population (15). Altogether, Sanger sequencing led to misclassification of approximately 6% of newly diagnosed and untreated patients harboring low-burden *TP53* mutations with a VAF ranging from 0.3 to 11% (15–19). Of note, a fraction of patients harbored low-burden mutations associated with high-burden mutations, revealing the intratumoral heterogeneity of these mutations and the complexity of the *TP53* clonal architecture.

The definition of minor clones often relies on the VAF threshold used to detect mutant alleles by Sanger sequencing, which is typically approximately 10–12%. This conventional threshold corresponds to the current recommendations published by ERIC in 2018, above which *TP53* mutations should be clinically reported. Mutations with VAFs below the threshold are considered low allele frequency, whereas VAFs above the threshold are of a high allele frequency. This recommendation is still currently applied due to technical difficulties in detecting low-burden mutations. However, with the wide generalization and feasibility of NGS sequencing on a routine basis, the threshold to report *TP53* mutations and hence to define minor clones is debated.

Indeed, below this arbitrary threshold of 10%, a wide range of *TP53* variants can be detected by NGS sequencing with high confidence until reaching a limit of detection as low as 0.3% VAF (corresponding to three mutant alleles in a background of 1,000 wild-type alleles) while respecting specific procedures and quality criteria. First, CLL lymphocyte population purity greater than 80% reduces the possibility of dilution in nontumoral DNA that could underestimate a very low-burden mutation. Second, sufficient DNA corresponding to >6,000 diploid genomes and a third high target read depth is required to detect a very low-burden mutation with VAF<1% (20). Finally, robust bioinformatic workflows were developed to call true variants distinguished from background error noise. However, despite the very high confidence of *TP53* variant detection by NGS sequencing, the limit of detection of these ultrasensitive technologies needs to be evaluated to distinguish true *TP53* variants from background sequencing noise to avoid misdiagnosing *TP53* unmutated patients as mutated. The sequencing background depends on sequencing technologies and library preparation, which differ in capture and amplicon-based processes (21, 22).

## Clonal Evolution of Low-Burden *TP53* Mutation After Chemotherapy

While *TP53* abnormalities account for approximately 10% of naïve-treatment patients, these abnormalities are found in greater than 40% of patients with fludarabine-refractory CLL, which highlights the phenomenon of clonal evolution of *TP53* mutation induced by chemotherapy (13). Despite the current recommendations that consider <10% of minor clones to be of uncertain significance, accumulating evidence based on longitudinal studies argues for the clinical relevance to report *TP53* minor clones (15, 18, 20, 23–25). NGS sequencing of serial samples before and after treatment has allowed characterization of the dynamics of the minor clones under treatment and demonstrated their biological and clinical relevance.

Longitudinal retrospective studies based on NGS sequencing of fludarabine relapsed/refractory *TP53* mutated patient samples showed that low-burden *TP53* mutations were detected early in the disease course and before the onset of chemotherapy. These pre-treatment samples were initially screened using Sanger sequencing, and mutations were missed due to the lack of sensitivity of the technique. Interestingly, longitudinal analysis indicated that the acquisition of *TP53* mutations clearly preceded karyotype evolution, which highlights the genetic instability related to the presence of a *TP53* mutation and its likely role in the development of a complex karyotype (24). It is widely accepted that chemotherapy plays a key role in driving the selection of clones carrying *TP53* mutations (26). Fludarabine is a purine analog that inhibits DNA synthesis in tumor cells. In the case of defects in the *TP53* pathway, CLL cells lose their capacity to stop cell division and to trigger apoptosis in response to chemotherapy. As a result, the mutation induces a fitness effect by conferring a growth and survival advantage to the low-burden *TP53* mutation, which expands under the selection pressure of chemotherapy (27). The fact that a given low-burden *TP53* variant detected at the time of treatment initiation is found at relapse after a fludarabine-based regimen clearly demonstrates that these minor clones are not sequencing artifacts and highlights the need to redefine this threshold for optimal clinical practice.

Finally, relative stability in the *TP53* variant allele frequency is observed in some patients as long as they are not treated with chemotherapy. This notion is particularly true for *IGHV*-mutated patients, which have a more indolent disease course and can show the persistence of the mutated clone for years (28–30). On the other hand, given the natural clonal evolution of the disease with time, *TP53* minor clones can also be acquired during the disease course, independent of any pressure of selection induced by chemotherapy. This finding justifies early and iterative screening for *TP53* abnormalities during follow-up and before each new line of treatment with a sensitive sequencing technique.

## Impact of Targeted Agents on Low-Burden *TP53* Mutations

Given that *TP53*-mutated patients can benefit from targeted therapies with improved remission duration, there is a need to evaluate the impact of these therapies on the evolution of the *TP53*-mutated clone. Data on the clonal evolution of low-burden *TP53* mutations upon targeted treatment are limited (23, 31). Malcikova et al. showed that upon the use of BcR or bcl2 inhibitors as a second line of treatment, the percentage of VAF in the residual lymphocytosis remains stable, which reflects the efficacy of these treatments on the mutated clones (23). Indeed, BcR and bcl2 inhibitors target the BcR signaling pathway and apoptosis, respectively, and therefore overcome the p53 pathway. However, the persistence of *TP53*-mutated clones after treatment shows the failure to eradicate the disease (32). In some progressive patients treated with targeted therapies, the major *TP53* mutated clone becomes minor. However, in these cases, mutations that confer resistance to ibrutinib (i.e., *BTK* mutation) or Venetoclax (i.e., *BCL2* mutations) are frequently found. In another longitudinal study including treatment-naïve and relapsed/refractory patients treated with BcR inhibitors, the dynamics of *TP53* mutated clones were complex. Most of the *TP53* mutations decreased or were undetectable, but one-third remained stable with no differences noted between low- or high-VAF clones. A small proportion of *TP53* mutations increased. After a prolonged follow-up of greater than 2 years, the overall stability of low-burden *TP53* mutations was noted, supporting the notion of the lack of specific positive selection of *TP53* mutations under conditions of ibrutinib treatment (31). Nevertheless, all these observations need to be confirmed in a cohort of patients treated with novel agents in the frontline setting. To date, this has not been explored within clinical studies, and data are preliminary, especially for bcl2 inhibitors.

## Low- and High-Burden *TP53* Mutations Have the Same Unfavorable Prognostic Impact

In most studies focusing on the clinical impact of *TP53* minor clones, an arbitrary threshold of 10–12% VAF was chosen to define patients with low- or high-burden *TP53*-mutated clones. Most studies conducted in untreated patients (15, 18, 20) showed that low-burden *TP53* mutations significantly reduced the OS compared to cases with unmutated *TP53* genes. Moreover, the impact on OS was the same for patients harboring minor clones or high-burden *TP53* mutations (**Table 1**). The clinical consequence of *TP53* mutations was similar when patients with low VAF were stratified into subclasses <1%, between 1% and 5% or 5% and 10%. Shorter OS was also confirmed when separately considering patients with single or multiple mutations classified as high VAF or low VAF (15, 20).

**Table 1:** Summary of the prognostic and predictive impact of *TP53* mutations evaluated in 6 studies in CLL.

Cohort			Total patients/patients treated during follow up	*TP53* mutated patients		OS (months)			Low burden threshold	Prognostic impact of low burden	Predictive impact of low burden
				High burden *TP53* mutations	Solely low burden *TP53* mutations	*TP53* wild type	*TP53* Mutated high burden	*TP53* Mutated low burden			
**Untreated patients**											
	Rossi 2014 (15)		309	28	15	75.1%*	34.6%*	46.3%	0.3–10%	p 0.0042	
	Nadeu 2016 (18)		405/208	28	16	82%*	54%*	64%*	0.3–12%	p 0.011	
	Bomben 2021 (20)		1,220	92	76	NR	60	80	0.4–10%	P <0.0001	
	Brieghel 2019 (33)		290/97	20	25	NR	60	NR	0.2–10%	NS	
**At the time of treatment**											
**First line (CIT)**	Rossi 2014 (15)		53	11	6	54.3%*	12.1%*	0%*	0.3–10%		p 0.017
	Bomben 2021 (20)		544	61	42	NR	47	62	0.4–10%		p <0.0001
	Brieghel 2019 (33)		61	7	10	72	26	14	0.2–10%		p 0.002
	Blakemore 2020 (34)		499	43	16	73	26.1	50.5	<12%		NS
	Malcikova 2021 (23)		511	59	82	68.4	21.6	40.8**	0.1–10%		p 0.0004
**2nd line Targeted Treatment**	Malcikova 2021 (23)		159	57	48	51.6	36	NR	0.1–10%		NS

*5 year OS.

**not receiving targeted Treatment.

NS, non-significative. The overall survival (OS) in subgroups of patients with TP53 wild type, low-burden, or high-burden TP53 mutations is indicated in months, or the 5 years OS rate* is reported. P value corresponds to a comparison of OS of TP53 low-burden mutated patients vs TP53 wild-type patients. NR, not reached; NS, not significant.

The presence of del(17p) and/or *TP53* mutations are parameters of the CLL-International Prognostic Index (CLL-IPI), which combines five parameters (age, clinical stage, *TP53*, *IGHV* mutational status, serum β2-microglobulin) to predict survival and time-to-first-treatment (TTFT) in CLL patients. However, the value of the VAF threshold used to consider *TP53* mutated considerably impacted this score. Indeed, revisited CLL-IPI combining both high- and low-VAF *TP53* mutations significantly better discriminated high-risk patients than standard CLL-IPI, which exclusively considered high-VAF *TP53* mutations (20, 23, 35). Therefore, minor clones should be considered to refine prognostication models.

Most studies evaluating the predictive impact of *TP53* mutations showed significantly reduced survival in CIT-treated patients harboring either low- or high-burden *TP53* mutations (15, 20, 23, 33). Clonal expansion is likely the main factor contributing to the inferior survival of CIT-treated patients with low-burden *TP53* mutations, as demonstrated by longitudinal studies comparing pre- and post-treatment samples showing that the mutation burden consistently increases at relapse (18, 20, 23). Furthermore, the risk of *TP53* mutation expansion beyond the current threshold of 10% in the first relapse was significantly higher for patients carrying mutations with VAF >1% than for those with VAF <1% (23). Additionally, very low clonal abundance cell populations (as low as 0.3%) are clinically relevant, as they are resistant to CIT, are positively selected and may become the dominant leukemic population at the time of relapse. Blakemore et al.’s (34) LRF CLL4 clinical trial could not demonstrate inferior survival associated with cases harboring <12% VAF *TP53* mutations but rather an intermediate-risk group, revealing heterogeneity among studies based on the patients included, the duration of follow-up, and the thresholds used.

Therefore, these observations strengthen the need to redefine the clinically relevant threshold of VAF, which better discriminates *TP53*-mutated patients who will benefit from a targeted therapy (15, 26, 36–38).

The literature on the impact of *TP53* minor clones on targeted therapies is less abundant. One study (23) showed that in a cohort of relapsed/refractory patients entering treatment with BcR and bcl2 inhibitors, OS in response to targeted treatment in *TP53*-mutated patients did not significantly differ from that of *TP53* wild type patients irrespective of VAF.

## Discussion

The main focus of this review was to demonstrate that low-burden *TP53* mutations have an impact on CLL survival. This review analyzing different retrospective and prospective CLL cohorts highlights the need to detect *TP53* mutations with highly sensitive NGS technology in a routine setting due to the clonal expansion of minor clones after CIT. NGS sequencing technology can detect low-burden *TP53* mutations that are as low as 0.3% over the background noise using specific bioinformatics pipelines. The clinical relevance of these low-burden mutations is evaluated as prognostic or predictive markers, and most of the studies identified that cases bearing low-burden *TP53* mutations (VAF <10%) experienced shorter OS similarly to cases with high-burden *TP53* mutations (VAF >10%) compared to patients harboring wild type *TP53*. These concordant observations highlight the need to redefine the threshold used to identify *TP53*-mutated cases, as these findings may have important implications in the setting of CLL treatment.

Low-VAF mutations showed the same molecular characteristics and distribution as high-VAF mutations, confirming that they are not sequencing artifacts. Moreover, the pathogenicity of these mutations was confirmed using different databases (IARC *TP53*, UMD database) (39, 40). Accordingly, in longitudinal studies, sequential samples from CIT-treated patients showed that minor clones were positively selected and became dominant at relapse, confirming that these low-burden mutations that initially occur in a minority of cells are true mutations that expand under selective pressure (26).

Focusing on studies designed to assess overall survival (OS) between cases harboring the wild-type *TP53* gene versus cases with low-burden *TP53* variant (15, 18, 20, 35), the frequencies of *TP53* mutation ranged from 10.6 to 27.5%, of which 26.8 to 45.2% cases exclusively harbored low-burden *TP53* mutations depending on the threshold used to discriminate between low- and high-VAF *TP53* mutations. Blakemore et al. failed to demonstrate a clinical impact of low-burden *TP53* mutations but identified an intermediate-risk group. These findings were probably due to the choice of an arbitrary threshold of 12% for discriminating low- and high-burden *TP53* mutations and a minimum VAF >1% (34).

The impact of *TP53* mutations on OS also depended on the composition of the cohort with different proportions of patients carrying mutated *IGHV* or 17p deletion or variable times to diagnosis. Indeed, newly diagnosed patients often harbor mutated *IGHV*, and *TP53* abnormalities may not have a negative impact on the indolent disease course (23, 28–30, 35). These observations suggest that *TP53* mutation testing should be performed exclusively before treatment. Conversely, Brieghel et al. demonstrated that neither high nor low burden *TP53* mutations at the time of CLL diagnosis influenced OS independently (35). Surprisingly, patients with 17p deletion had an inferior outcome, and only the subgroup of patients with high-burden *TP53* mutations and unmutated *IGHV* demonstrated an inferior OS. This discrepancy may be explained by the composition of the cohort and the more indolent nature of the disease for the patients included. The frequency of 17p deletion was only 2.4%, whereas *TP53* mutations without 17p deletions were more frequent (10.7%). Furthermore, the proportion of newly diagnosed *TP53*-mutated patients with unmutated *IGHV* genes was low (32%) as compared to 57% (18) and 35.5% (15).

Given that NGS technology can detect low-burden *TP53* mutations at levels as low as 0.3%, should this limit of detection be used as a threshold to identified patients with *TP53* mutations? One study further stratified patients based on a 5% VAF threshold and observed shortened survival only for mutations with 5–10% VAF but not for mutations with 1–5% VAF. Interestingly, the subgroup carrying mutations with <1% VAF showed significantly shortened OS. In addition, the risk of a rapid expansion of the clone to greater than 10% in the first relapse after CIT treatment was higher for patients carrying mutations with >1% VAF than for those with <1% VAF (23). These results suggest that a >1% VAF threshold could be clinically relevant.

Further standardization (41) and bioinformatics development (42) may be necessary to identify the background noise at each position of the *TP53* gene to validate very low-burden mutations (as low as 0.3%).

Hence, there is a need to harmonize the methodologies used to detect minor clones and minimal requirements for the standardized assessment of such clones. An ERIC (European research initiative on CLL http://www.ericll.org/) multicenter study on the prognostic and predictive impact of low-burden *TP53* mutations is in progress with three phases: 1) compare results among laboratories performing NGS analysis of *TP53* mutations in CLL with a detection limit of ≤1% VAF, 2) assess the prognostic and predictive impact of low-VAF *TP53* variants in patients entering first-line treatment, and 3) re-evaluate the cut-off for reporting of *TP53* variants in CLL and, if needed, to update recommendations on minor *TP53* variant detection, validation, and reporting. Forty-one laboratories participated in the 1st phase of the study and analyzed the same samples with low-VAF *TP53* mutations. The collected results show that the 2% VAF cut-off could be reproducibly applied for the planned multicenter study on the clinical significance of low-VAF *TP53* variants (43). The collection of clinical and biological data from a consecutive cohort of patients, namely, both wild-type and mutated *TP53* CLL entering 1st-line therapy, is currently in progress to re-evaluate the cut-off for reporting *TP53* variants.

## Author Contributions

All authors listed have made a substantial, direct, and intellectual contribution to the work and approved it for publication.

## Conflict of Interest

The authors declare that the research was conducted in the absence of any commercial or financial relationships that could be construed as a potential conflict of interest.

## Publisher’s Note

All claims expressed in this article are solely those of the authors and do not necessarily represent those of their affiliated organizations, or those of the publisher, the editors and the reviewers. Any product that may be evaluated in this article, or claim that may be made by its manufacturer, is not guaranteed or endorsed by the publisher.
